# GATA4 Variants in Individuals With a 46,XY Disorder of Sex Development (DSD) May or May Not Be Associated With Cardiac Defects Depending on Second Hits in Other DSD Genes

**DOI:** 10.3389/fendo.2018.00142

**Published:** 2018-04-04

**Authors:** Idoia Martinez de LaPiscina, Carmen de Mingo, Stefan Riedl, Amaia Rodriguez, Amit V. Pandey, Mónica Fernández-Cancio, Nuria Camats, Andrew Sinclair, Luis Castaño, Laura Audi, Christa E. Flück

**Affiliations:** ^1^Endocrinology and Diabetes Research Group, BioCruces Health Research Institute, Cruces University Hospital, CIBERDEM, CIBERER, UPV-EHU, Barakaldo, Spain; ^2^Pediatric Endocrinology, Diabetology and Metabolism, Department of Pediatrics, Inselspital, Bern University Hospital, University of Bern, Bern, Switzerland; ^3^Pediatric Endocrinology, Diabetology and Metabolism, Department of BioMedical Research, Inselspital, Bern University Hospital, University of Bern, Bern, Switzerland; ^4^Pediatric Endocrinology, La Fe Pediatric University Hospital, Valencia, Spain; ^5^Division of Pediatric Pulmology, Allergology, and Endocrinology, St. Anna Children’s Hospital, Department of Pediatrics, Medical University of Vienna, Vienna, Austria; ^6^Pediatric Endocrinology Section, Cruces University Hospital, BioCruces Health Research Institute, CIBERDEM, CIBERER, UPV/EHU, Barakaldo, Spain; ^7^Growth and Development Research, Pediatric Endocrinology Unit, Vall d’Hebron Research Institute (VHIR), CIBERER, Instituto de Salud Carlos III, Barcelona, Spain; ^8^Department of Paediatrics, Murdoch Children’s Research Institute, University of Melbourne, The Royal Children’s Hospital, Melbourne, VIC, Australia

**Keywords:** disorder of sexual development, DSD, GATA4, congenital heart defects, oligogenic, 46,XY DSD

## Abstract

Disorders of sex development (DSD) consist of a wide range of conditions involving numerous genes. Nevertheless, about half of 46,XY individuals remain genetically unsolved. *GATA4* gene variants, mainly related to congenital heart defects (CHD), have also been recently associated with 46,XY DSD. In this study, we characterized three individuals presenting with 46,XY DSD with or without CHD and *GATA4* variants in order to understand the phenotypical variability. We studied one patient presenting CHD and 46,XY gonadal dysgenesis, and two patients with a history of genetically unsolved 46,XY DSD, also known as male primary hypogonadism. Mutation analysis was carried out by candidate gene approach or targeted gene panel sequencing. Functional activity of GATA4 variants was tested *in vitro* on the CYP17 promoter involved in sex development using JEG3 cells. We found two novel and one previously described *GATA4* variants located in the N-terminal zinc finger domain of the protein. Cys238Arg variant lost transcriptional activity on the CYP17 promoter reporter, while Trp228Cys and Pro226Leu behaved similar to wild type. These results were in line with bioinformatics simulation studies. Additional DSD variations, in the *LRP4* and *LHCGR* genes, respectively, were identified in the two 46,XY individuals without CHD. Overall, our study shows that human *GATA4* mutations identified in patients with 46,XY DSD may or may not be associated with CHD. Possible explanations for phenotypical variability may comprise incomplete penetrance, variable sensitivity of partner genes, and oligogenic mechanisms.

## Introduction

Disorders of sex development (DSD) are defined as congenital conditions, in which development of chromosomal, gonadal, or anatomical sex is atypical (1). 46,XY DSD includes disorders in male gonad determination and differentiation, androgen biosynthesis or action, and anti-Müllerian hormone (AMH) synthesis or action (1). In the last two decades, numerous genes have been found to cause 46,XY DSD (2). In about 40% of 46,XY DSD individuals, the underlying genetic cause still remains unsolved. GATA4 has been more recently implicated in 46,XY DSD.

The GATA family of transcription factors consists of six members, three expressed in hematopoietic stem cells (GATA1–3) and three in tissues derived from mesoderm and endoderm, including heart, gonad, lung, liver, and gut (GATA4–6). GATA factors regulate tissue-specific gene expression either alone or in cooperation with other factors (3, 4). GATA proteins have two zinc fingers (ZNI and ZNII), which are highly conserved while the sequences of the amino-terminal and carboxyl-terminal domains exhibit lower similarities (5, 6). While the C-terminal zinc finger region is required for the DNA recognition and binding, and the N-terminal zinc finger region contributes to the stability, both zinc fingers are necessary for protein–protein interactions with other transcription factors (4, 7).

The human *GATA4* gene on chromosome 8p23.1 encodes an essential transcription factor for the developing gonad (8) and heart (9, 10). Gata4-null mice die due to severe abnormalities in heart tube formation and ventral morphogenesis (9, 10). Gata4 expression and function seems required for normal testicular and genital development (11–14). Human GATA4 interacts with several proteins, including NR5A1, WT1, and FOG2 to regulate the expression of sex determining genes *SRY, SOX9*, and *AMH*, as well as genes involved in sex differentiation such as *StAR, CYP17A1, CYP19A1, INHA*, and *HSD3B2* (15–18).

In humans, *GATA4* haploinsufficiency has been described in patients with different forms of congenital heart defects (CHD) since 1999 (OMIM_600576). So far more than 120 *GATA4* gene variants have been associated with cardiac defects (HGMD^®^ professional 2017, accessed October 2017), while only four studies reported mutations related to a 46,XY DSD phenotype (19–22).

Overall, *GATA4* gene variations seem more likely to result in CHD than DSD, but the reason for this is unclear. And the fact that some individuals with GATA4 haploinsufficiency have a 46,XY DSD phenotype only remains similarly unexplained.

We, therefore, characterized three additional individuals presenting with 46,XY DSD and two novel and one previously described *GATA4* variants. They presented with or without CHD, but additional variants in two other genes were found that likely contributed to the DSD phenotype.

## Materials and Methods

### Ethical Approval

Written informed consent was obtained from all subjects and their family members at the respective hospitals involved. The study was approved by the local ethical committees, namely the ethical boards of the La Fe University Hospital, Valencia, Spain, the St. Anna Children’s Hospital, Vienna, Austria, as well as the ethics committee for clinical research of Euskadi (CEIC-E), Spain.

### Genetic Analysis

Genomic DNA was isolated from peripheral blood leukocytes and analyzed for genetic alterations causing DSD using different approaches.

*Case 1* was first studied by a candidate gene approach. *GATA4* gene segments corresponding to the 5′UTR region, the 6 coding exons and their flanking intronic sequences were amplified by PCR using specific primers [Table S1 in Supplementary Material; (21)]. The PCR products were sequenced using the BigDye Terminator v3.1 Cycle Sequencing Kit on an automated ABI PRISM 3100 Genetic Analyzer (Applied biosystems, Foster City, CA, USA). Obtained sequences were analyzed and compared with the wild-type (wt) published reference sequence (RefSeq NM_002052) using SeqScape Software v. 2.5 (Applied Biosystems). The DNA sample was in addition studied on a sequencing panel (TruSight One Sequencing Panel, Illumina, San Diego, CA, USA) containing 4,813 disease-associated genes, including 94 well-known candidate genes for DSD 46,XY and 46,XX.

*Case 2* was examined on the target gene panel for DSD by Haloplex technology (Agilent, Santa Clara, CA, USA). This system allowed to simultaneously sequence 64 diagnostic genes for DSD and 967 candidate genes (19).

*Case 3* was analyzed by a customized Ion Ampliseq panel (ThermoFisher Scientific, Waltham, MA, USA) comprising coding and flanking regions of 48 DSD genes. Analysis was performed according to the manufacturer’s instructions.

Of note, GATA4 variants identified by panel analysis were verified by Sanger sequencing. Parents and affected family members were tested to establish the mode of inheritance.

Identified *GATA4* sequence variants were then evaluated for their possible functional significance *in silico* using prediction software programs, including SIFT,^1^ Provean,^2^ PolyPhen-2,^3^ MutationTaster,^4^ MutPred,^5^ and SNPs&GO.^6^

In all three cases, the involved clinician informed the patient about the genetic results, including information on pathogenic, probably pathogenic, and uncertain findings with regard to the DSD/genital phenotype. Incidental findings were not made.

### *In Vitro* Functional Studies

Human placental JEG3 cells (300222^7^), human adrenal NCI-H295R cells (ATCC CRL-2128^8^), and non-steroidogenic human embryonic kidney HEK293 cells (ATCC CRL-1573) were cultured as described (18, 23) and used for functional assays. Promoter luciferase reporter vector −227CYP17A1_Δluc was available from previous work (18). Promoter reporter vectors pGL3AMH and pGL3SRY were cloned by PCR amplifying the fragments of −1,067/+32 *SRY* and −951/+115 *AMH* from human genomic wt DNA and inserting the fragments into pGL3 basic (Promega, Dübendorf, Switzerland). Vectors were verified by direct sequencing.

Human wt GATA4 cDNA was inserted into the mammalian pCMV expression vector. GATA4 mutations c.684A, c.712C, and c.677T were generated by site-directed mutagenesis using specific primers and the QuickChange protocol by Stratagene (Agilent technologies Inc., Santa Clara, CA, USA). We also constructed the previously described c.661A GATA4 variant for comparison (21). Mutant plasmids were confirmed by direct sequencing.

For functional studies, cells were cultured on 12-well plates and transiently transfected with wt or mutant GATA4 vectors and a CYP17 promoter reporter using a calcium phosphate transfection protocol (Invitrogen, ThermoFisher Scientific). 48 h after transfection, cells were washed and lysed, before luciferase activity was measured with the Dual-Luciferase^®^ Reporter (DLR™) Assay system (Promega AG, Wallisellen, Switzerland) on a Veritas microplate Luminometer reader (Turner Bio systems Luminometer and Software by Promega). Specific *Firefly* luciferase readings were standardized against *Renilla* control readings and results expressed as relative luciferase units. Results are shown as the mean ± SEM of four independent experiments, all performed in duplicate. Data were statistically analyzed using the Student’s *t* test. A *p*-value ≤ 0.05 was considered significant.

### Protein Structure Analysis

Since the structure of GATA4 protein is not known, a hybrid homology model of the protein was made using YASARA (24) and AMBER. Three rounds of PSI-BLAST were performed with the GATA4 amino acid sequence (NP_002043) to build a position-specific scoring matrix, and then a search of protein structure database was conducted to find the templates for model generation. Structures of GATA3 (PDB # 4HC9 and 4HC7) and GATA1 (PDB # 3VD6 and 3VEK) were found to be best templates for making the GATA4 protein model. Using each template separately several models were made. Three loops from amino acid sequences NLDMFDDFSE, KNLNKSKTPA, and LIKPQRRLSASRRVGL needed to be modeled separately and joined in the structure. Side chains of modeled amino acids were optimized by screening of rotamer libraries and molecular dynamics (MD) simulations. Best quality parts from different models were then combined to generate a hybrid model, which had increased coverage and accuracy compared to individual models. *In silico* mutagenesis was performed to create the GATA4 mutations described in this report (25), which were then further refined by brief MD simulations of 500 ps. All models were then subjected 500 steps of steepest descent and simulated annealing minimizations using AMBER14 force filed and TIP3P water model (26, 27), and then subjected to 25 ns of explicit solvent MD simulations at 310 K. Structural analysis was performed after the MD simulation systems were stable. All illustrations were prepared with PyMOL^9^ and rendered as ray-traced images using PovRay.^10^

## Results

### Cases Reports of Three 46,XY DSD Patients Harboring GATA4 Gene Variations

#### Case 1

This “female” newborn was referred after birth because of ambiguous genitalia (Table 1). She presented with a small vulva, slight clitoral hypertrophy, labia were fused in posterior raphe and gonads were palpable in inguinal canal. Karyotype was 46,XY. Ultrasound and pelvic MRI showed a rudimentary uterus. Biochemical and hormonal studies at presentation and during follow-up revealed more or less normal values (compared to male age-controls), only undetectable and low AMH was remarkable (Table 2). She was also seen by a pediatric cardiologist for a heart murmur and found to have a complex CHD, which was not detected in prenatal screening (Table 1). At 40 days of life, surgical closure of the ventricular septal defect was performed. It was then noted that she also had congenital compression of the left bronchus leading to an asymmetry in the caliber of the pulmonary branches with a hypoplastic left branch. During follow-up over 3 years, she showed some signs of developmental delay. She is reared as a girl.

**Table 1:** Clinical and genetic characterization of three 46,XY DSD patients harboring variants in the *GATA4* gene.

Case	Gender assigned	Phenotype					Genotype		
			
		Genital anatomy at initial presentation	Müllerian ducts	Gonadectomy	CHD	Syndromic features	Karyotype	*GATA4* gene variant	Other DSD genes
1	F	Clitoral hypertrophy, fused labia with posterior raphe, gonads palpable in inguinal canal	Rudimentary uterus	No. But, planned with cardiac surgery	Yes	Developmental delay: sitting 12 months, crawling 18 months, walking 24 months; at 3 years no language skills, signs of autism	46,XY	c.712T>C; p.Cys238Arg	None

2	M	Micropenis, hypospadias, bilateral cryptorchidism	No	No	No	No	46,XY	c.684G>C; p.Trp228Cys	*LRP4* (c.5660C>G; p.Ser1887Cys)

3	M	Micropenis, bilateral cryptorchidism (inguinal)	No	No	No	Severe obesity	46,XY	c.677C>T; p.Pro226Leu	*LHCGR* (c.1660C>T; p.Arg554Stop)

*All sequence information about GATA4 gene is based on NM_002052. LRP4 and LHCGR information is based on NM_002334 and NM_000233, respectively*.

*CHD, congenital heart defects; LHCGR, luteinizing hormone/choriogonadotropin receptor; LRP4, LDL receptor-related protein 4*.

**Table 2:** Biochemical characterization of three 46,XY DSD patients harboring variants in the *GATA4* gene.

Case	Adrenal function							Gonadal function					
		
	Age at evaluation	ACTH (pg/ml)	Cortisol (μg/dl)	11-Deoxycortisol (ng/ml)	17OHP (ng/ml)	DHEA-S (ng/ml)	Androstenedione (ng/ml)	T (ng/ml)		FSH (U/l)	LH (U/l)	Estradiol (pg/ml)	AMH (ng/ml)
		
		Basal	Basal	Basal	Basal	Basal	Basal	Basal	After hCG	Basal	Basal	Basal	Basal
1	2 days	**243.0**	11.3	11.4	4.4	**1,660**		1.30		0.2	0.1	59	**<0.14**
	7 days	36.7	**1.9**										
	14 days									**19.3**	3.4	42	
	3.5 years					123	<0.3	<0.02		**14.1**	<0.1	<5.0	**4.4**

2	3 months							0.92		**38**	**12.3**		
	7 months							0.11	1.87				
	10 years	16	8.6		0.31	2.41	0.55	0.21		**14.9**	**0.9**	13	14.4

3	11 years							**1.30**		**63.3**	**14**		
	14 years							**1.30**		**36.7**	**18.5**		
	15 years							5.9^a^		1.5^a^	**<0.5^a^**		
	21 years							11.8^a^		9.5^a^	4.0^a^		

*Laboratory test values outside the normal range of age and chromosomal sex are given in bold*.

*ACTH, adrenocorticotropic hormone; 17OHP, 17-hydroxyprogesterone; DHEA-S, dehydroepiandrosterone sulfate; T, testosterone; FSH, follicle stimulating hormone; LH, luteinizing hormone; AMH, anti-Müllerian hormone*.

*^a^Under testosterone replacement treatment*.

#### Case 2

Patient 2 presented micropenis with hypospadias and cryptorchidism at birth (Table 1). His karyotype was 46,XY. At 3 months of age, his gonadotropins were elevated at a testosterone of 0.92 ng/ml (Table 2). At 7 months, spontaneous descensus of the left testis occurred, whereas the right testis was found inguinal. Stimulation test with hCG resulted in a normal rise of testosterone. He was treated for micropenis with testosterone (3 mg × 25 mg monthly), which increased phallic size from 2.0 to 3.5 cm. At 17 months, right-sided orchidopexy was performed including biopsy. Histology revealed a sertoli-cell only phenotype; spermatogonia were not visible. Tubules appeared immature exhibiting a compressed, lumen-less structure. At follow-up at 10 years of age, testicular sonography revealed multiple calcifications with a testis size of 0.5 ml on the right and 0.8 ml on the left. The boy was still prepubertal with a small phallus of 3 cm and a hypoplastic scrotum. Biochemically, luteinizing hormone (LH), follicle stimulating hormone (FSH), and testosterone were already on the rise. AMH and adrenal steroids were in the normal, age- and sex-appropriate range. Apart from the development of his external genitalia, the boy has been healthy and psychomotor development is normal. He has no CHD.

Family history is remarkable for the following: the boy’s mother is healthy with no fertility problems, but was diagnosed with a mitral valve prolapse in adulthood requiring no medical intervention. According to cardiologists, mitral valve prolapse is often seen with aging and does not qualify as CHD. The maternal uncle had a similar history as the patient with micropenis and bilateral cryptorchidism at birth. Orchidopexy and testosterone treatment for micropenis were performed in early childhood. Pubertal onset occurred spontaneously and no testosterone treatment was required. At 18 years of age, he had markedly elevated gonadotropins (LH 25.8 IU/l; FSH 53.8 IU/l) in the presence of a low-normal testosterone. Testicular volume was 3 ml. Azoospermia was ascertained at 23 years and subsequent testicular biopsy revealed sertoli-cell only syndrome. During the following years, testosterone levels decreased continuously and AMH became undetectable. He is otherwise healthy, without signs of a CHD.

#### Case 3

This patient was noted at birth to have micropenis and bilateral cryptorchidism, but no action was taken (Table 1). At 11 years of age he still presented micropenis (3.5 cm), testes were non-palpable and severe obesity (BMI 36.6, +6.4 SD) was observed. His gonadotropins were elevated with low testosterone. Karyotype was 46,XY. MRI showed both testes in inguinal canal. At 14.3 years, replacement testosterone treatment was started with gradual increasing doses. After treatment, LH suppression and normalization of FSH was observed. At 18 years, azoospermia was found. At recent follow-up at 21 years of age, he presented with severe obesity (BMI 44.9, +6.8 SD) and short stature (156.1 cm). Penis (8 cm) was buried in subcutaneous fat and left testis was in scrotum (0.5 ml). Biochemically, he presented normal gonadotropins with a testosterone of 11.8 ng/ml under treatment. Ultrasound showed an ovoid structure (17 mm × 16 mm) in right inguinal canal corresponding to atrophic testis. Currently, laparoscopy is planned to investigate the right testis and biopsy both to assess the malignancy risk. Echocardiography revealed no CHD.

### Genetic Characterization of Three Novel *GATA4* Gene Variants

We found novel *GATA4* gene variants in three patients presenting with 46,XY DSD at birth.

In case 1, direct analysis by Sanger sequencing revealed a T to C change in heterozygosis at position 712 (c.712T>C; p.Cys238Arg) in exon 3 of GATA4. Both parents were studied and did not carry the variant, thus this GATA4 variant is a *de novo* change in the patient.

In case 2, DSD targeted gene panel sequencing identified a heterozygous GATA4 c.684G>C nucleotide change, which is predicted to result in a p.Trp228Cys alteration. This variant has been previously listed in a publication by Eggers et al. reporting on DSD panel diagnostics (19). Remarkably, the patients’ mother presenting with a mitral valve prolapse, and his maternal uncle having a similar history of micropenis and cryptorchidism, were both carriers of the same heterozygous GATA4 variant.

In case 3, a heterozygous C to T transition located at c.677 of the coding GATA4 DNA sequence predicted to replace a proline with leucine at position p.226 (c.677C>T; p.Pro226Leu) was found by DSD targeted panel sequencing. The same heterozygous variant was also found in the healthy mother.

**Figure 1:**
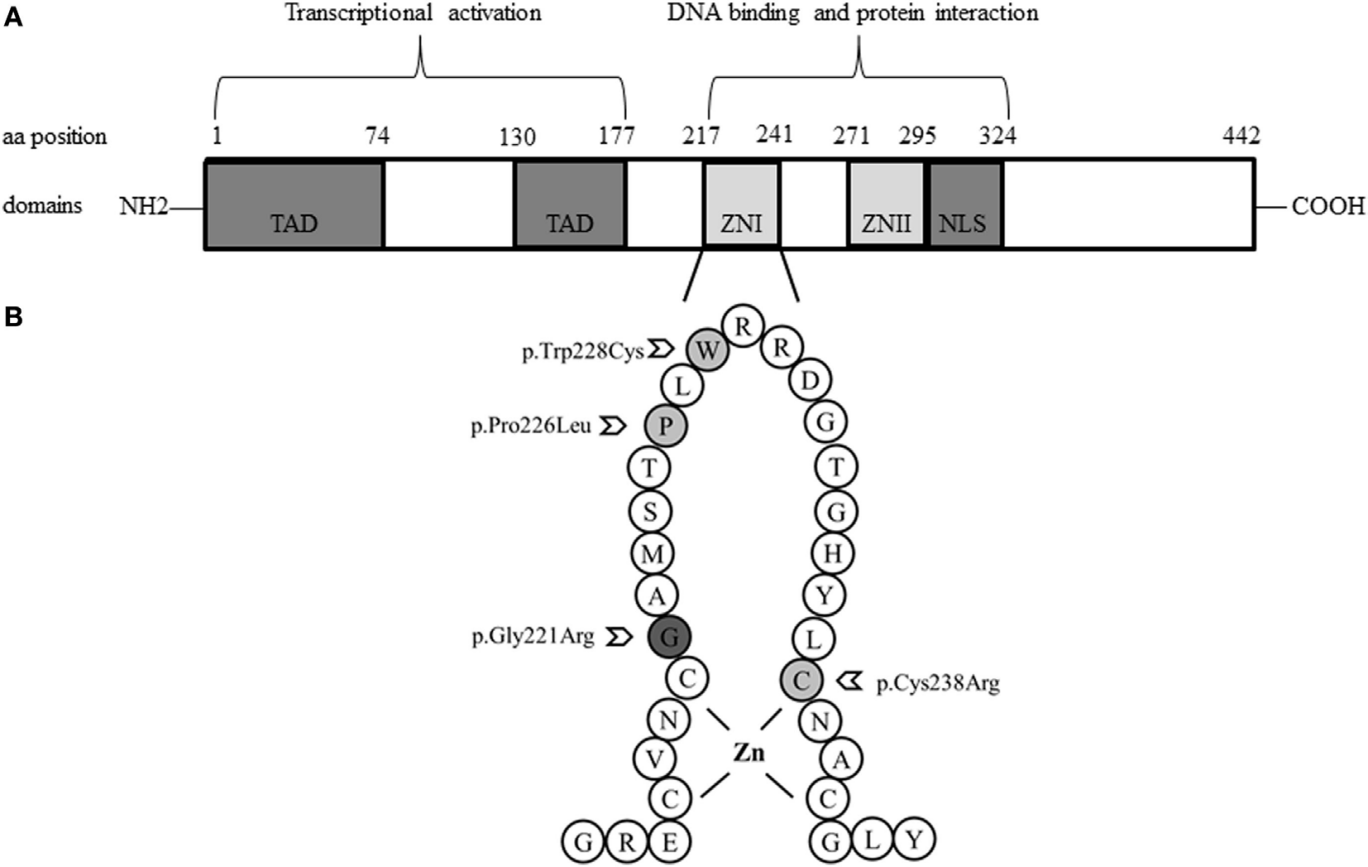
Schemes of the structure of the GATA4 protein and localization of studied variations. **(A)**. GATA4 contains two distinct zinc finger domains (ZNI and ZNII) and a C-terminal nuclear localization sequence (NLS), which consists of a DNA-binding domain and a protein–protein interaction domain. Transcriptional activation domains (TAD) are located in the N-terminus. **(B)**. Close-up loop of the ZNI domain of the C-terminus, where all GATA4 variations described so far in patients with a 46,XY phenotype are located: Gly221Arg, Pro226Leu, Trp228Cys, and Cys238Arg.

**Figure 2:**
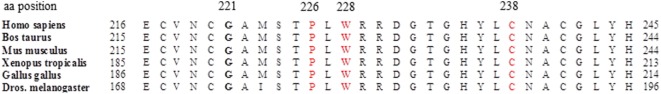
Multiple alignment of parts of the GATA4 protein sequences across species. Localization of the newly identified human aa variants are given in red and seem highly conserved across species. The localization of the so far only GATA4 mutation reported to cause a 46,XY DSD phenotype (p.Gly221Arg) is given in bold and its localization is also highly conserved.

Looking at these GATA4 variants, it is remarkable that all are located in the N-terminal zinc finger domain of the GATA4 protein (Figure 1), where the previously described variant p.Gly221Arg (found in a patient with 46,XY DSD) is also located (21). Prediction software programs and comparison of GATA4 proteins across species revealed that Cys238, Trp228, and Pro226, and the surrounding region are highly conserved (Figure 2). Therefore, the identified GATA4 gene variants were predicted as potentially pathogenic.

### Additional (DSD) Gene Variations Identified by Next Generation Sequencing (NGS)

In case 1, additional targeted exome sequencing of 4,813 disease-causing genes, containing 94 genes related to DSD, did not reveal any further sequence variant. In cases 2 and 3, without CHD, DSD targeted gene sequencing identified two additional gene variations. Case 2 revealed a novel, heterozygous C to G change at position 5660 (c.5560C>G; p.Ser1887Cys) of the LDL receptor-related protein 4 (*LRP4*) gene (OMIM 604270). This gene variant was also found in the mother and the maternal uncle. In case 3, a heterozygous change of cytosine to thymine was found at location 1660 of the *LHCGR* gene (OMIM 152790). This mutation changes codon 554 from arginine to a stop codon (c.1160C>T; p.Arg554Stop) and has been previously reported (28). The mother did not carry this luteinizing hormone/choriogonadotropin receptor (LHCGR) mutation.

### *In Vitro* Functional Studies of Novel GATA4 Variants

**Figure 3:**
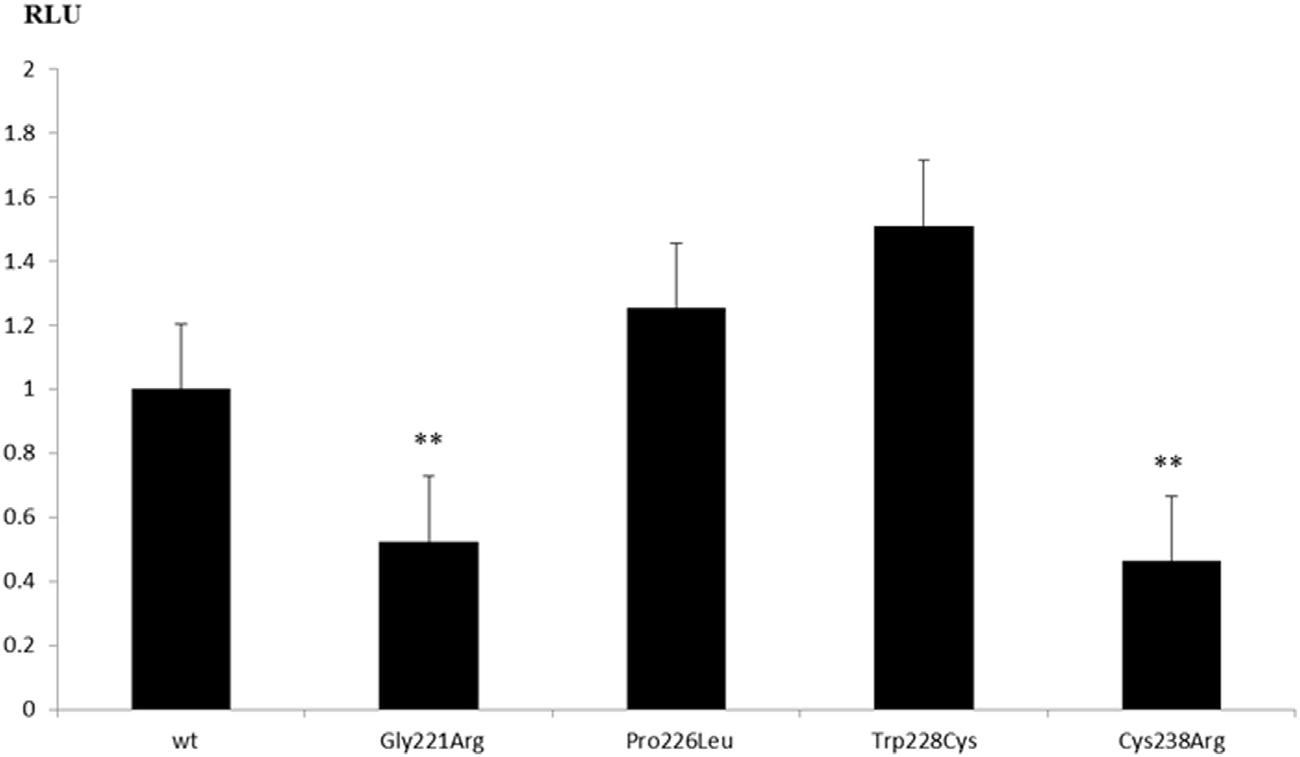
Transcriptional activity of GATA4 variants on the *CYP17A1* promoter. Human placental JEG3 cells were transfected with a CYP17-promoter luciferase reporter construct, and the activity of wild-type (wt) and mutant GATA4 to trans-activate the promoter was tested using the Promega Dual Luciferase readout system. Results are shown as the mean ± SEM of four independent experiments, all performed in duplicate. **p-Value ≤ 0.01.

To test the transcriptional activity of identified GATA4 variants, we constructed mammalian expression vectors of wt and mutant GATA4 and tested them on three different promoters that have been described being regulated by GATA4, namely the AMH, SRY, and CYP17 promoters. For these studies, we used different cell systems (HEK293, NCI-H295R, and JEG3), but found that only JEG3 cells transfected with the CYP17 promoter revealed consistent results for comparing wt to mutant GATA4. We found that GATA4 variant Cys238Arg lost transcriptional activity (Figure 3) similar to the previously described Gly221Arg mutant (21). By contrast, GATA4 variants Trp228Cys and Pro226Leu activated the CYP17 promoter similar to wt.

### *In Silico* GATA4 Protein Structure-Function Analysis

**Figure 4:**
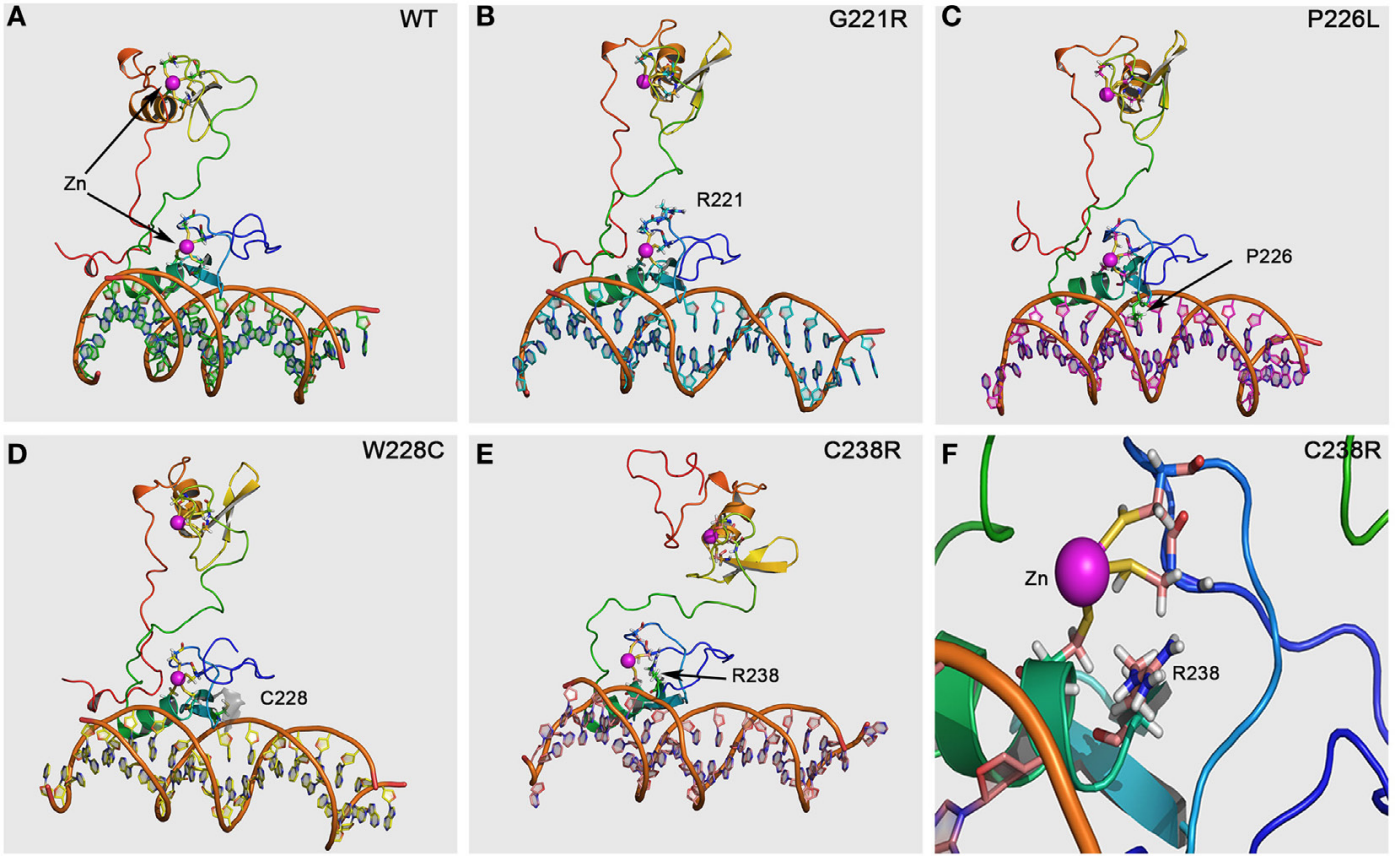
GATA4 protein model and prediction of effects by mutations. The GATA4 protein model was made using the structures of GATA1 and GATA3 available in the protein structure database. After performing a structure-based search using Phi-BLAST, two different structures of GATA1 and GATA3 were selected and used for model building as described in Section “Materials and Methods.” **(A)** Structural of WT GATA4 showing the Zn coordination and binding to DNA. **(B)** The Gly221Arg mutation is predicted to cause disruption of Zn binding and protein instability, resulting in a loss of function mutation. **(C)** The mutation Pro226Leu is in the nucleotide interaction interface and was predicted to alter the recognition of promoter sequences for binding to GATA4. This can lead to variable effects with some promoters showing on effect while other could be affected. **(D)** The mutation Trp228Cys was also predicted to cause variable effects due to its proximity to DNA-binding interface of GATA4. Inclusion of extra cysteine was not predicted to cause the disruption of Zn binding as Cys228 in the mutant was far away from the Zn binding site. **(E)** The mutation Cys238Arg was predicted to cause loss of Zn binding, leading to an instable and non-functional GATA4 protein. **(F)** A close-up of the Cys238Arg mutation showing the loss of Zn coordination to cysteine due to introduction of the arginine, which could not bind the Zn molecule to stabilize the protein. A severe loss of function effect was predicted for the Cys238Arg mutation.

Using the structures of GATA1 and GATA3 available in the protein structure database, we created a protein structure model of GATA4. GATA4 was modeled with dual zinc atoms, which were coordinated with cysteines (Figure 4). One Zn atom is bound to cysteines 217, 220, 238, and 241, while the second Zn atom is bound to cysteines 272, 275, 293, and 296. Mutation of any of these cysteine molecules as well as neighboring residues is expected to result in loss of Zn binding. Among the mutations studied in this report, we found the mutations Gly221Arg and Cys238Arg to cause loss of Zn binding leading to an unstable protein with predicted loss of function (Figure 4). The mutations Pro226Leu and Trp228Cys are involved in interaction with DNA and their effects were predicted to be variable. Since the core structure of the GATA4 protein is not expected to be affected by these two mutations, it is the interactions with different DNA fragments that is predicted to be changed. As we have observed in case of SF1 mutations, the mutations causing a change in the interaction with DNA can have variable effects (23). While interaction and activation of one partner sequence can be affected and may cause full or partial loss of activation, the newly created nucleotide recognition element due to mutations could interact and activate another promoter sequence, resulting in altered gene expression. In case of Gly221Arg, loss of interaction with FOG2 protein has been reported. Structural analysis of this mutation shows a likelihood of unstable protein due to loss of Zn binding and therefore, an alteration in the interaction with GATA4 binding partners is expected.

## Discussion

We characterized three human GATA4 sequence variations found in three individuals with a 46,XY DSD phenotype with and without CHD. These variants were all missense changes and were present in heterozygous form. NGS revealed additional likely disease causing variants in two other genes (so-called second hits), namely *LRP4* or *LHCGR*, in two patients with a 46,XY DSD phenotype without CHD.

In both mice and men, GATA4 is expressed in the somatic cell population of the developing heart and gonads and plays an essential role in their development (8–10). Human GATA4 mutations associated with cardiac malformations are described in numerous patients (Table S2 in Supplementary Material) and are located throughout the gene in the coding and non-coding sequence including 3′- and 5′-UTR (29–33). They comprise single nucleotide variants as well as deletions and duplications, which lead to missense/nonsense mutations or splicing errors and thus altered proteins or protein expression. Studies of the pathogenesis of CHD have reported that mutations in the GATA4 gene decrease the ability to *trans*activate target genes or fail to interact with proteins involved in heart development (34, 35). Moreover, it has been recently demonstrated that common variants in a region of GATA4 3′UTR contribute to the risk of CHD by changing posttranscriptional gene regulation at the level of miRNA (36). Altogether, these studies show that *GATA4* gene mutations contribute to the susceptibility of CHD. A list of these numerous GATA4 mutations discovered in patients with CHD so far is provided in Table S2 in Supplementary Material. For a more comprehensive review of the role of GATA4 in CHD we refer to Ref. (31).

By contrast, the few GATA4 missense mutations found in 46,XY DSD individuals with or without CHD are all located in the N-terminal zinc finger domain, which is responsible for DNA binding and interaction with cofactors (4, 7).

Functional characterization of GATA4 variants with respect to the 46,XY DSD phenotype has only been performed for the p.Gly221Arg mutation so far (21). *In vitro* studies revealed that p.Gly221Arg lacked DNA binding, had impaired transactivation activity on the AMH promoter, and failed to bind cofactor FOG2. Functional testing of three GATA4 variants identified in 46,XY DSD individuals of our study showed similarly disruptive effect for the missense mutation p.Cys238Arg, but no effect on transactivation activity on the CYP17 promoter for GATA4 variants p.Pro226Leu and pTrp228Cys. While all these variants are conserved across species (Figure 2) and located in the N-terminal zinc finger domain of GATA4 (Figure 1), only Gly221 and Cys238 are close to Zn binding sites. The Gly221 is not directly involved in Zn binding but is situated next to Cys220 which binds the Zn atom, and therefore, the mutation Gly221Arg will disrupt the Zn binding, leading to a non-functional GATA4. The Cys238 binds Zn and its mutation to arginine leads to loss of Zn binding (Figure 4).

GATA4 regulates the expression of multiple genes coding for hormones or components of the steroidogenic pathway during testis development and function. In Gata4^ki^ mice with p.Val217Gly mutation interaction of Gata4 with cofactor Fog is abrogated, and consequently animals display anomalies of testis development (12–14). Moreover, GATA4 functionally interacts with NR5A1 in Sertoli cell cultures to positively regulate the expression of AMH, and therefore, it has been reported that mutations in NR5A1 may cause 46,XY DSD due to lack of interaction with GATA (15). No gonadal involvement is mostly detected in families with *GATA4* mutations and isolated CHD, possibly because some of the variants retain some DNA-binding activity and exhibit different degrees of transcriptional activation on gonadal promoters and thus, remain able to synergize with NR5A1. In the present study, the p.Cys238Arg mutation was found in a patient with a complex CHD, genital ambiguity, and persistent Müllerian ducts, which led to female gender assignment. We propose that cysteine to arginine change in position 238 of GATA4 lacks activity to bind DNA reducing the transactivation of AMH critically.

By contrast, variants p.Pro226Leu and pTrp228Cys found in cases 2 and 3 did not affect CYP17 promoter activity. These individuals had a less severe 46,XY DSD phenotype, were raised as males, and had no evidence of heart anomalies. White et al. (22) described a 35 kb deletion immediately 3′ UTR of the *GATA4* gene in a patient presenting with complete gonadal dysgenesis (GD). Additionally, Harrison et al. (20) screened patients with 46,XY DSD/GD with array CGH and found an infant presenting a deletion of 0.22 Mb upstream *GATA4* in chromosome 8p23.1. Same deletion was found in his healthy mother and his maternal grandmother, who had CHD. The authors proposed that the identified rearrangements obviously do not affect the coding sequence of GATA4, and may, therefore, not manifest with CHD, but rather disrupt regulatory elements controlling gene expression essential in the developing gonad (22). Thus, the phenotypic variability could be explained by genetic modifiers (37, 38). In addition, GATA4 regulates multiple (gonadal) promoters to variable degrees, rendering some promoters more sensitive to haploinsufficiency than others (15–18, 21, 39). Structural changes caused by both the p.Pro226Leu and pTrp228Cys variations were not predicted to be disruptive and core GATA4 structure was not altered. Since the changes were in the DNA interaction sites, it is expected that both p.Pro226Leu and pTrp228Cys mutations could have altered binding and activation of some of GATA4 interaction partners and could also bind to other promoters and potentially change the transcription of several other genes.

In fact, we found segregating genetic variants besides GATA4 in cases 2 and 3 using NGS. In one 46,XY DSD subject without CHD, a heterozygote variant in *LRP4* gene was found. Mutations in *LRP4* have been related to the Cenani–Lenz syndactyly syndrome and disruption of canonical WNT/beta-catenin signaling (OMIM 604270), which is not only important in bone formation but also in sexual development (40).

In our other 46,XY DSD patient without CHD, a heterozygote mutation in *LHCGR* gene was found together with the GATA4 variant. The same inactivating *LHCGR* mutation was previously reported in 46,XY DSD and 46,XX primary amenorrhea (28). LHCGR is essential for fetal differentiation of the neutral external genitalia into the male phenotype. Inactivating, homozygous, or compound heterozygous mutations of *LHCGR* cause resistance to hormonal stimulation and varying degree of Leydig cell hypoplasia in 46,XY subjects (41–43). Looking at the 5′UTR of *LHCGR*, several GATA sites are present suggesting that GATA4 may regulate this gene. Therefore, combined heterozygocity for *GATA4* and *LHCGR* variants in our patient may explain the 46,XY DSD phenotype.

Finally, phenotypical variability with same heterozygous *GATA4* mutation (p.Gly221Arg) observed within same family manifesting with either 46,XY DSD or CHD only, indicates that there might be incomplete penetrance (21). Interestingly, the same observation was made in mice heterozygote for a *Gata4* deletion (44).

In conclusion, detailed characterization of three new 46,XY DSD patients with and without CHD harboring heterozygous *GATA4* missense mutations in comparison to previously reported patients revealed possible explanations for phenotypical variability. Thereby incomplete penetrance, variable sensitivity, and oligogenic mechanisms may be considered. Interestingly, “double hits” combining a *GATA4* variant with *LHCGR* or *LRP4* variants have been found in two individuals with a 46,XY DSD phenotype only.

## Ethics Statement

Approval for this study was obtained from ethical committees related to: (1) Pediatric Endocrinology, Hospital Infantil La Fe, Av. Campanar 21, 46009 Valencia, Spain. (2) Division of Pediatric Pulmology, Allergology, and Endocrinology, St. Anna Children’s Hospital, Department of Pediatrics, Medical University of Vienna, Währinger Gürtel 18-20, 1090 Vienna, Austria. (3) Pediatric Endocrinology Section, Cruces University Hospital, BioCruces Health Research Institute, CIBERDEM, CIBERER, UPV/EHU, Plaza de Cruces 12, 48903, Barakaldo, Spain.

## Author Contributions

Study design: IM, CM, LA, and CF. Clinical work-up: CM, SR, AR, and LC. Genetic work-up: IM, MF-C, NC, AS, and LA. *In vitro* studies: IM, CF. Bioinformatic studies: AP. Data analysis: IM, AP, and CF. Data interpretation: IM, AP, LA, and CF. Manuscript preparation: IM, CM, LA, and CF. Manuscript approval: all.

## Conflict of Interest Statement

The authors report no conflict of interest in this work.

## Footnotes

^1^http://sift.jcvi.org/ (Accessed: March, 2017).

^2^http://provean.jcvi.org/index.php (Accessed: March, 2017).

^3^http://genetics.bwh.harvard.edu/pph2/ (Accessed: March, 2017).

^4^http://www.mutationtaster.org/ (Accessed: March, 2017).

^5^http://mutpred.mutdb.org/ (Accessed: March, 2017).

^6^https://snps-and-go.biocomp.unibo.it/snps-and-go/ (Accessed: March, 2017).

^7^http://www.clsgmbh.de/ (Accessed: April, 2017).

^8^http://www.lgcstandards-atcc.org (Accessed: April, 2017).

^9^www.pymol.org (Accessed: October, 2017).

^10^www.povray.org (Accessed: October, 2017).
