# Supplementary material for: GATA4 Variants in Individuals With a 46,XY Disorder of Sex Development (DSD) May or May Not Be Associated With Cardiac Defects Depending on Second Hits in Other DSD Genes
Source: Front Endocrinol (Lausanne). 2018 Apr 4;9:142. doi: 10.3389/fendo.2018.00142 (PMC5893726; doi:10.3389/fendo.2018.00142)
Supplement: Supplementary file 1 [file table_1.PDF]

Suppl Table 1. Newly designed primers for sequencing exons 3, 5 and 7 of *GATA4*. Other exons were amplified using primers published in Ref. 21.

PRIMER	Forward	Reverse
**Exon 3**	GGCTCTGAATGTGATACCTG	CAAGGCCACTAGCTTTTCTG
**Exon 5**	GAGAGATTGCTTAGGTGTTG	TTCTTAGGCACTCTGAGGG
**Exon 7**	CACCCTCCCCAGCCTAGAC	CTCCTTCTTTGCTATCCTCC
