# Supplementary material for: GATA4 Variants in Individuals With a 46,XY Disorder of Sex Development (DSD) May or May Not Be Associated With Cardiac Defects Depending on Second Hits in Other DSD Genes
Source: Front Endocrinol (Lausanne). 2018 Apr 4;9:142. doi: 10.3389/fendo.2018.00142 (PMC5893726; doi:10.3389/fendo.2018.00142)
Supplement: Supplementary file 2 [file table_2.PDF]

Supplemental Table 2. Reported variants in the *GATA4* gene associated with cardiac defects (HGMD® professional 2017, as of October 2017).

	Nucleotide change; protein change	Domain	Reported phenotype	Genetic test used	Functionally studied in a model system	Categorization of data1	Reference
Missense variants	c.17C>T; p.Ala6Val	TAD1	VSD	Candidate gene	No	1	Zhang W (2008) (1)
	c.25G>C; p.Ala9Pro	TAD1	TOF	Candidate gene	Yes	1	Yang YQ (2013a) (2)
	c.46G>T; p.Gly16Cys	TAD1	Lone AF	Candidate gene	Yes	1	Jiang JQ (2011) (3)
	c.62G>T; p.Gly21Val	TAD1	ASD	Candidate gene	Yes	1	Liu XY (2011) (4)
	c.82C>G; p.His28Asp	TAD1	Lone AF	Candidate gene	Yes	1	Jiang JQ (2011) (3)
	c.82C>T; p.His28Tyr	TAD1	CSD	Candidate gene	No	1	Chen Mw (2010a) (5)
	c.106C>T; p.Pro36Ser	TAD1	ASD	Candidate gene	No	1	Yang YQ (2013b) (6)
	c.112T>G; p.Tyr38Asp	TAD1	AF	Candidate gene	Yes	1	Wang J (2012) (7)
	c.115G>T; p.Val39Leu	TAD1	DCM	Candidate gene	Yes	1	Li J (2014) (8)
	c.127C>T; p.Arg43Trp	TAD1	VSD	Candidate gene	Yes	1	Yang YQ (2012a) (9)
	c.151C>G; p.Leu51Val	TAD1	TOF	Candidate gene	Yes	1	Yang YQ (2013a) (2)
	c.155C>T; p.Ser52Phe	TAD1	ASD	Candidate gene	No	1	Hirayama- Yamada K (2005) (10)
	c.164A>G; p.Gln55Arg	TAD1	VSD	Candidate gene	No	1	Yang YQ (2012b) (11)
	c.196G>A; p.Ala66Thr	TAD1	VSD	Candidate gene	No	1	Chen Mw (2010a) (5)
	c.206G>A; p.Gly69Asp	TAD1	VSD	Candidate gene	Yes	2	Butler TL (2010) (12)
	c.209G>C; p.Ser70Thr	TAD1	AF	Candidate gene	Yes	1	Yang YQ (2011) (13)
	c.221C>A; p.Ala74Asp	TAD1	PS	Candidate gene	Yes	1	Wang E (2013) (14)
	c.278G>C; p.Gly93Ala		ASD	Candidate gene	No	2	Tomita- Mitchell A (2007) (15)
	c.286G>A; p.Gly96Arg		VSD	Candidate gene	No	1	Yang YQ (2012b) (11)
	C.307C>G; p.Pro103Ala		AF	Candidate gene	Yes	1	Wang J (2012) (7)
	c.343G>T; p.Gly115Trp		ASD	WES	Yes	1	LaHaye S (2016) (16)
	c.431C>T; p.Ala144Val	TAD2	CHD	Candidate gene	No	1	Yoshida A (2016) (17)
	c.448G>T; p.Gly150Trp	TAD2	TOF	Candidate gene	Yes	1	Wang E (2013) (14)
	c.479G>C; p.Ser160Thr	TAD2	AF	Candidate gene	Yes	1	Yang YQ (2011) (13)
	c.487C>T; p.Pro163Ser	TAD2	ECD	Candidate gene	No	2	Rajagopal SK (2007) (18)
	c.488C>G; p.Pro163Arg	TAD2	VSD	Candidate gene	Yes	2	Butler TL (2010) (12)
	c.500C>A; p.Ala167Asp	TAD2	CTD	Candidate gene	Yes	2	Liu Y (2017) (19)
	c.569A>G; p.His190Arg		ASD	Candidate gene	No	1	Yang YQ (2013b) (6)
	c.578C>A; Pro193His		CHD	Candidate gene	No	1	Shaker O (2017) (20)
	c.590A>G; p.Asn197Ser		VSD	Candidate gene	No	1	Yang YQ (2012b) (11)
	c.628G>A; p.Asp210Asn		CHD	Candidate gene	Yes	2	Wang E (2013) (14)
	c.648G>C; p.Glu216Asp		TOF	Candidate gene	Yes	1	Nemer G (2006) (21)
	c.677C>A; p.Pro226Gln	ZNI	DCM	Candidate gene	Yes	1	Li J (2014) (8)
	c.740T>C; p.Met247Thr		AF	Candidate gene	No	1	Posch MG (2010) (22)
	c.749T>A; p.Ile250Asn		VSD	Candidate gene	Yes	2	Wang E (2013) (14)
	c.784T>G; p.Ser262Arg		ASD	Candidate gene	No	1	Yang YQ (2013b)
	c.788C>G; p.Arg263Gly		VSD	Candidate gene	No	1	Xiong F (2013) (23)
	c.812G>C; p.Cys271ser	ZNII	DCM	Candidate gene	Yes	1	Li RG (2013) (24)
	c.835A>T; p.Thr279Ser	ZNII	DCM	Candidate gene	Yes	1	Li J (2014) (8)
	c.839C>T; p.Thr280Met	ZNII	ASD	Candidate gene	No	1	Chen Y (2010b) (25)
	c.851G>A; p.Arg284His	ZNII	ASD	Candidate gene	No	1	El Malti R (2016) (26)
	c.854A>G; p.Asn285Ser	ZNII	TOF	Candidate gene	Yes	1	Yang YQ (2013a) (2)
	c.871G>C; p.Val291Leu	ZNII	DCM	Candidate gene	Yes	1	Zhao L (2014) (27)
	c.886G>T; p.Gly296cys	NLS	ASD	Candidate gene	No	1	Rajagopal SK (2007) (18)
	c.886G>C; p.Gly296Arg	NLS	VSD	Candidate gene	Yes	1	Wang J (2011) (28)
	c.886G>A; p.Gly296Ser	NLS	ASD	Candidate gene	Yes	1	Garg V (2003) (29)
	c.899A>C; p.Lys300Thr	NLS	ASD	Candidate gene + WES	No	1	Chen J (2016) (30)
	c.928A>G; p.Met310Val	NLS	ASD	Candidate gene	No	1	Chen Y (2010) (31)
	c.931C>T; p.Arg311Trp	NLS	TOF	Candidate gene	Yes	1	Zhang X (2016) (32)
	c.946C>G; p.Gln316Glu	NLS	ASD	Candidate gene	No	1	Tomita- Mitchell A (2007)(15)
	c.955A>G; p.Lys319Glu	NLS	ASD+PS	Candidate gene	No	1	Xiang R (2014) (33)
	c.958C>T; p.Arg320Trp	NLS	ASD	Candidate gene	Yes	1	D'Amato E (2010) (34)
	c.989C>G; p.Thr330Arg		PTA	Candidate gene	Yes	1	Kodo K (2012) (35)

	c.1037C>T; p.Ala346Val		ECD	Candidate gene	No	1	Rajagopal SK (2007) (18)
	c.1057G>A; p.Ala353Thr		TOF	Candidate gene	Yes	1	Wang E (2013) (14)
	c.1075G>A; p.Glu359Lys		VSD	Candidate gene	No	1	Zhang W (2008) (1)
	c.1079A>G; p.Glu360Gly		VSD	Candidate gene	Yes	1	Wang E (2013) (14)
	c.1129A>G; p.Ser377Gly		CHD	Candidate gene	Yes	2	Wang E (2013) (14)
	c.1180C>G; p.Pro394Ala		ASD/VSD/ASD+VSD	Candidate gene	No	1	Dinesh SM (2011) (36)
	c.1196T>G; p.Val399Gly		ASD	Candidate gene	No	1	Yang YQ (2013b) (6)
	c.1207C>A; p.Leu403Met		HRV	Candidate gene	No	1	Rajagopal SK (2007) (18)
	c.1211A>G; p.Lys404Arg		VSD	Candidate gene	No	1	Yang YQ (2012b) (11)
	c.1220C>A; p.Pro407Gln		TOF	Candidate gene	No	1	Zhang W (2008) (1)
	c.1232C>T; p.Ala411Val		VSD	Candidate gene	No	2	Tomita- Mitchell A (2007) (15)
	c.1273G>A; p.Asp425Asn		ASD/ TOF	Candidate gene	No	2	Tomita- Mitchell A (2007) (15)
	c.1286G>C; p.Ser429Thr		VSD	Candidate gene	No	1	Zhang W (2008) (1)
	c.1295T>C; p.Leu432Ser		PDA	Candidate gene	No	1	Dinesh SM (2011) (36)
	c.1306C>T; p.His436Tyr		CSD	Candidate gene	Yes	1	Chen Mw (2010a) (5)
	c.1310G>C; p.Gly437Ala		BAV	Candidate gene	No	2	Bonachea EM (2014) (37)
	c.1325C>T; p.Ala442Val		VSD	Candidate gene	No	1	Zhang W (2008) (1)
Splicing variants	c.-458+5G>A		TOF	Candidate gene	No	3	Mattapally S (2015) (38)
	c.909+5G>A		CHD	Candidate gene	No	1	Yoshida A (2016) (17)
	c.998-269G>T		VSD	Candidate gene	No	3	Mattapally S (2015) (38)
	c.998-219G>T		VSD	Candidate gene	No	3	Mattapally S (2015) (38)
	c.997+56C>A		TOF	Candidate gene	No	3	Mattapally S (2015) (38)
Regulatory sequence variants	C>T +12, relative to transcription initiation site		ASD	Candidate gene	No	3	Mattapally S (2015) (38)
	T>C -930, relative to transcription initiation site		VSD	Candidate gene	Yes	1	Wu G (2012) (39)
	C>A -853, relative to transcription initiation site		VSD	Candidate gene	Yes	1	Wu G (2012) (39)
	C>T -435, relative to transcription initiation site		VSD	Candidate gene	Yes	1	Wu G (2012) (39)
	G>T -348, relative to transcription initiation site		VSD	Candidate gene	Yes	1	Wu G (2012) (39)
	G>C -6, relative to initiation codon		ASD	Candidate gene	Yes	1	Mohan RA (2014) (40)
	A>T + 119, relative to termination codon		CHD	Candidate gene	No	2	Reamon-Buettner SM (2007) (41)
	C>G +1521, relative to termination codon		CoHD	Candidate gene	Yes	4	Sabina S (2013) (42)
Insertions and deletions	c.353delC		CHD	Candidate gene	Yes	2	Tong YF (2016) (43)
	NG_008177:g.4690Gdel		VSD	Candidate gene	No	1	Wu G (2012) (39)
	c.139_141delTCC	TAD1	VSD	Candidate gene	No	1	Zhang W (2008) (1)
	c.1074delC		ASD	Candidate gene	No	1	Okubo A (2004) (44)
	c.1075delG		CHD	Candidate gene	Yes	1	Garg V (2003) (29)
	c.341_342insA		ASD	Candidate gene	No	1	Hamanoue H (2009) (45)
	c.366_368dupCGC		TOF	Candidate gene	No	1	Zhang W (2008) (1)
	c.369_374dupTGCCGC		VSD	Candidate gene	No	1	Zhang W (2008) (1)
	Del <10.8kb including non-coding exon 1		VSD	MLPA	No	2	El Malti R (2016) (26)
	Del1.1Mb, includes entire gene + others		AVSD	Array	No	2	Priest JR (2016) (46)
	Del 104.5kb, includes entire gene		TOF	Array	No	1	Glessner JT (2014) (47)
	Del 124.633, includes part gene + other		VSD	Array	No	1	Warburton D (2014) (48)
	Del 127 kb, includes entire gene + other		VSD	Array	No	1	Keitges EA (2013) (49)
	Del 24900kb, includes entire gene		CHD	Array	No	1	Geng J (2014) (50)
	Del 3.3Mb, includes entire gene		LVNC	Array	No	2	Blinder JJ (2011) (51)
	Del 3.6Mb, includes entire gene + others		AVSD	Array	No	2	Priest JR (2016) (46)
	Del 304kb, includes entire gene + others		ASD	Array	No	2	Tomita-Mitchell A (2012) (52)
	Del 315kb, includes entire gene + others		CHD	Array	No	1	Keitges EA (2013) (49)
	Del 3844kb, includes entire gene + others		A-V canal	Array	No	2	Tomita-Mitchell A (2012) (52)
	Del 4456kb, includes entire gene + others		VSD	Array	No	2	Tomita-Mitchell A (2012) (52)
	Del 4500kb, includes entire gene		CHD	Array	No	1	Geng J (2014) (50)
	Del 5.5Mb, includes entire gene + others		CHD	Array	No	2	Guimiot F (2013) (53)
	Del 5.7Mb, includes entire gene + others		CHD	Array	No	2	Guimiot F (2013) (53)
	Del 5200kb, includes entire gene		CHD	Array	No	1	Geng J (2014) (50)
	Dup 11500kb, includes entire gene		CHD	Array	No	1	Geng J (2014) (50)
	Dup 1438kb, includes entire gene + others		HLHS	Array	No	2	Tomita-Mitchell A (2012) (52)
	Dup 3.7Mb, includes entire genes		TOF	Array	No	2	Osoegawa K (2014) (54)
	Dup 3800kb, includes entire gene		CHD	Array	No	1	Geng J (2014) (50)
	Dup 4087.9kb, includes entire gene		CHD	Array	No	1	Glessner JT (2014) (47)
	Dup 4463.2kb, includes entire gene		TOF	Array	No	1	Glessner JT (2014) (47)

[1]Categorization according to HGMD® professional 2017: 1, disease causing mutation; 2, disease causing mutation?; 3, disease- associated polymorphism; 4, disease- associated polymorphism supporting functional evidence
Abbreviations: AF, atrial fibrillation; ASD: atrial septal defect; AVSD: atrioventricular septal defect; BAV: Bicuspid aortic valve; CoHD, coronary Heart Disease; CSD, congenital cardiac septal defect; CTD, Conotruncal Heart Defects; DCM, Dilated cardiomyopathy ; Del, deletion; Dup, duplication; ECD, endocardial cushion defect; HLHS, Hypoplastic left heart syndrome; HRV, hipoplastic right ventricle; LVNC, Left ventricular noncompaction; OFT, Cardiac outflow tract; PDA, patent ductus arteriosus; PS, pulmonary stenosis; PTA, persistent truncus arteriosus; TOF, Tetralogy of Fallot; VSD, Ventricular septal defect

References

1. **Zhang W, Li X, Shen A, Jiao W, Guan X, Li Z** 2008 GATA4 mutations in 486 Chinese patients with congenital heart disease. European journal of medical genetics 51:527-535
2. **Yang YQ, Gharibeh L, Li RG, Xin YF, Wang J, Liu ZM, Qiu XB, Xu YJ, Xu L, Qu XK, Liu X, Fang WY, Huang RT, Xue S, Nemer G** 2013 GATA4 loss-of-function mutations underlie familial tetralogy of fallot. Human mutation 34:1662-1671
3. **Jiang JQ, Shen FF, Fang WY, Liu X, Yang YQ** 2011 Novel GATA4 mutations in lone atrial fibrillation. International journal of molecular medicine 28:1025-1032
4. **Liu XY, Wang J, Zheng JH, Bai K, Liu ZM, Wang XZ, Liu X, Fang WY, Yang YQ** 2011 Involvement of a novel GATA4 mutation in atrial septal defects. International journal of molecular medicine 28:17-23
5. **Chen MW, Pang YS, Guo Y, Pan JH, Liu BL, Shen J, Liu TW** 2010 GATA4 mutations in Chinese patients with congenital cardiac septal defects. Pediatric cardiology 31:85-89
6. **Yang YQ, Wang J, Liu XY, Chen XZ, Zhang W, Wang XZ** 2013 Mutation spectrum of GATA4 associated with congenital atrial septal defects. Archives of medical science : AMS 9:976-983
7. **Wang J, Sun YM, Yang YQ** 2012 Mutation spectrum of the GATA4 gene in patients with idiopathic atrial fibrillation. Molecular biology reports 39:8127-8135
8. **Li J, Liu WD, Yang ZL, Yuan F, Xu L, Li RG, Yang YQ** 2014 Prevalence and spectrum of GATA4 mutations associated with sporadic dilated cardiomyopathy. Gene 548:174-181
9. **Yang YQ, Li L, Wang J, Liu XY, Chen XZ, Zhang W, Wang XZ, Jiang JQ, Liu X, Fang WY** 2012 A novel GATA4 loss-of-function mutation associated with congenital ventricular septal defect. Pediatric cardiology 33:539-546
10. **Hirayama-Yamada K, Kamisago M, Akimoto K, Aotsuka H, Nakamura Y, Tomita H, Furutani M, Imamura S, Takao A, Nakazawa M, Matsuoka R** 2005 Phenotypes with GATA4 or NKX2.5 mutations in familial atrial septal defect. American journal of medical genetics. Part A 135:47-52
11. **Yang YQ, Wang J, Liu XY, Chen XZ, Zhang W, Wang XZ, Liu X, Fang WY** 2012 Novel GATA4 mutations in patients with congenital ventricular septal defects. Medical science monitor : international medical journal of experimental and clinical research 18:CR344-350
12. **Butler TL, Esposito G, Blue GM, Cole AD, Costa MW, Waddell LB, Walizada G, Sholler GF, Kirk EP, Feneley M, Harvey RP, Winlaw DS** 2010 GATA4 mutations in 357 unrelated patients with congenital heart malformation. Genetic testing and molecular biomarkers 14:797-802
13. **Yang YQ, Wang MY, Zhang XL, Tan HW, Shi HF, Jiang WF, Wang XH, Fang WY, Liu X** 2011 GATA4 loss-of-function mutations in familial atrial fibrillation. Clinica chimica acta; international journal of clinical chemistry 412:1825-1830
14. **Wang E, Sun S, Qiao B, Duan W, Huang G, An Y, Xu S, Zheng Y, Su Z, Gu X, Jin L, Wang H** 2013 Identification of functional mutations in GATA4 in patients with congenital heart disease. PloS one 8:e62138
15. **Tomita-Mitchell A, Maslen CL, Morris CD, Garg V, Goldmuntz E** 2007 GATA4 sequence variants in patients with congenital heart disease. Journal of medical genetics 44:779-783
16. **LaHaye S, Corsmeier D, Basu M, Bowman JL, Fitzgerald-Butt S, Zender G, Bosse K, McBride KL, White P, Garg V** 2016 Utilization of Whole Exome Sequencing to Identify Causative Mutations in Familial Congenital Heart Disease. Circulation. Cardiovascular genetics 9:320-329
17. **Yoshida A, Morisaki H, Nakaji M, Kitano M, Kim KS, Sagawa K, Ishikawa S, Satokata I, Mitani Y, Kato H, Hamaoka K, Echigo S, Shiraishi I, Morisaki T** 2016 Genetic mutation analysis in Japanese patients with non-syndromic congenital heart disease. Journal of human genetics 61:157-162
18. **Rajagopal SK, Ma Q, Obler D, Shen J, Manichaikul A, Tomita-Mitchell A, Boardman K, Briggs C, Garg V, Srivastava D, Goldmuntz E, Broman KW, Benson DW, Smoot LB, Pu WT** 2007 Spectrum of heart disease associated with murine and human GATA4 mutation. Journal of molecular and cellular cardiology 43:677-685
19. **Liu Y, Li B, Xu Y, Sun K** 2017 Mutation Screening of Gata4 Gene in CTD Patients Within Chinese Han Population. Pediatric cardiology 38:506-512

20. **Shaker O, Omran S, Sharaf E, G AH, Mashaly M, N EAG** 2017 A novel mutation in exon 1 of GATA4 in Egyptian patients with congenital heart disease. Turkish journal of medical sciences 47:217-221
21. **Nemer G, Fadlalah F, Usta J, Nemer M, Dbaibo G, Obeid M, Bitar F** 2006 A novel mutation in the GATA4 gene in patients with Tetralogy of Fallot. Human mutation 27:293-294
22. **Posch MG, Boldt LH, Polotzki M, Richter S, Rolf S, Perrot A, Dietz R, Ozcelik C, Haverkamp W** 2010 Mutations in the cardiac transcription factor GATA4 in patients with lone atrial fibrillation. European journal of medical genetics 53:201-203
23. **Xiong F, Li Q, Zhang C, Chen Y, Li P, Wei X, Zhou W, Li L, Shang X, Xu X** 2013 Analyses of GATA4, NKX2.5, and TFAP2B genes in subjects from southern China with sporadic congenital heart disease. Cardiovascular pathology : the official journal of the Society for Cardiovascular Pathology 22:141-145
24. **Li RG, Li L, Qiu XB, Yuan F, Xu L, Li X, Xu YJ, Jiang WF, Jiang JQ, Liu X, Fang WY, Zhang M, Peng LY, Qu XK, Yang YQ** 2013 GATA4 loss-of-function mutation underlies familial dilated cardiomyopathy. Biochemical and biophysical research communications 439:591-596
25. **Chen Y, Mao J, Sun Y, Zhang Q, Cheng HB, Yan WH, Choy KW, Li H** 2010 A novel mutation of GATA4 in a familial atrial septal defect. Clinica chimica acta; international journal of clinical chemistry 411:1741-1745
26. **El Malti R, Liu H, Doray B, Thauvin C, Maltret A, Dauphin C, Goncalves-Rocha M, Teboul M, Blanchet P, Roume J, Gronier C, Ducreux C, Veyrier M, Marcon F, Acar P, Lusson JR, Levy M, Beyler C, Vigneron J, Cordier-Alex MP, Heitz F, Sanlaville D, Bonnet D, Bouvagnet P** 2016 A systematic variant screening in familial cases of congenital heart defects demonstrates the usefulness of molecular genetics in this field. European journal of human genetics : EJHG 24:228-236
27. **Zhao L, Xu JH, Xu WJ, Yu H, Wang Q, Zheng HZ, Jiang WF, Jiang JF, Yang YQ** 2014 A novel GATA4 loss-of-function mutation responsible for familial dilated cardiomyopathy. International journal of molecular medicine 33:654-660
28. **Wang J, Fang M, Liu XY, Xin YF, Liu ZM, Chen XZ, Wang XZ, Fang WY, Liu X, Yang YQ** 2011 A novel GATA4 mutation responsible for congenital ventricular septal defects. International journal of molecular medicine 28:557-564
29. **Garg V, Kathiriya IS, Barnes R, Schluterman MK, King IN, Butler CA, Rothrock CR, Eapen RS, Hirayama-Yamada K, Joo K, Matsuoka R, Cohen JC, Srivastava D** 2003 GATA4 mutations cause human congenital heart defects and reveal an interaction with TBX5. Nature 424:443-447
30. **Chen J, Qi B, Zhao J, Liu W, Duan R, Zhang M** 2016 A novel mutation of GATA4 (K300T) associated with familial atrial septal defect. Gene 575:473-477
31. **Chen Y, Han ZQ, Yan WD, Tang CZ, Xie JY, Chen H, Hu DY** 2010 A novel mutation in GATA4 gene associated with dominant inherited familial atrial septal defect. The Journal of thoracic and cardiovascular surgery 140:684-687
32. **Zhang X, Wang J, Wang B, Chen S, Fu Q, Sun K** 2016 A Novel Missense Mutation of GATA4 in a Chinese Family with Congenital Heart Disease. PloS one 11:e0158904
33. **Xiang R, Fan LL, Huang H, Cao BB, Li XP, Peng DQ, Xia K** 2013 A novel mutation of GATA4 (K319E) is responsible for familial atrial septal defect and pulmonary valve stenosis. Gene
34. **D'Amato E, Giacopelli F, Giannattasio A, D'Annunzio G, Bocciardi R, Musso M, Lorini R, Ravazzolo R** 2010 Genetic investigation in an Italian child with an unusual association of atrial septal defect, attributable to a new familial GATA4 gene mutation, and neonatal diabetes due to pancreatic agenesis. Diabetic medicine : a journal of the British Diabetic Association 27:1195-1200
35. **Kodo K, Nishizawa T, Furutani M, Arai S, Ishihara K, Oda M, Makino S, Fukuda K, Takahashi T, Matsuoka R, Nakanishi T, Yamagishi H** 2012 Genetic analysis of essential cardiac transcription factors in 256 patients with non-syndromic congenital heart defects. Circulation journal : official journal of the Japanese Circulation Society 76:1703-1711
36. **Dinesh SM, Lingaiah K, Savitha MR, Krishnamurthy B, Narayanappa D, Ramachandra NB** 2011 GATA4 specific nonsynonymous single-nucleotide polymorphisms in congenital heart disease patients of Mysore, India. Genetic testing and molecular biomarkers 15:715-720
37. **Bonachea EM, Zender G, White P, Corsmeier D, Newsom D, Fitzgerald-Butt S, Garg V, McBride KL** 2014 Use of a targeted, combinatorial next-generation sequencing approach for the study of bicuspid aortic valve. BMC medical genomics 7:56
38. **Mattapally S, Nizamuddin S, Murthy KS, Thangaraj K, Banerjee SK** 2015 c.620C>T mutation in GATA4 is associated with congenital heart disease in South India. BMC medical genetics 16:7
39. **Wu G, Shan J, Pang S, Wei X, Zhang H, Yan B** 2012 Genetic analysis of the promoter region of the GATA4 gene in patients with ventricular septal defects. Translational research : the journal of laboratory and clinical medicine 159:376-382
40. **Mohan RA, van Engelen K, Stefanovic S, Barnett P, Ilgun A, Baars MJ, Bouma BJ, Mulder BJ, Christoffels VM, Postma AV** 2014 A mutation in the Kozak sequence of GATA4 hampers translation in a family with atrial septal defects. American journal of medical genetics. Part A 164A:2732-2738
41. **Reamon-Buettner SM, Cho SH, Borlak J** 2007 Mutations in the 3'-untranslated region of GATA4 as molecular hotspots for congenital heart disease (CHD). BMC medical genetics 8:38

42. **Sabina S, Pulignani S, Rizzo M, Cresci M, Vecoli C, Foffa I, Ait-Ali L, Pitto L, Andreassi MG** 2013 Germline hereditary, somatic mutations and microRNAs targeting-SNPs in congenital heart defects. Journal of molecular and cellular cardiology 60:84-89
43. **Tong YF** 2016 Mutations of NKX2.5 and GATA4 genes in the development of congenital heart disease. Gene 588:86-94
44. **Okubo A, Miyoshi O, Baba K, Takagi M, Tsukamoto K, Kinoshita A, Yoshiura K, Kishino T, Ohta T, Niikawa N, Matsumoto N** 2004 A novel GATA4 mutation completely segregated with atrial septal defect in a large Japanese family. Journal of medical genetics 41:e97
45. **Hamanoue H, Rahayuningsih SE, Hirahara Y, Itoh J, Yokoyama U, Mizuguchi T, Saitsu H, Miyake N, Hirahara F, Matsumoto N** 2009 Genetic screening of 104 patients with congenitally malformed hearts revealed a fresh mutation of GATA4 in those with atrial septal defects. Cardiology in the young 19:482-485
46. **Priest JR, Osoegawa K, Mohammed N, Nanda V, Kundu R, Schultz K, Lammer EJ, Girirajan S, Scheetz T, Waggott D, Haddad F, Reddy S, Bernstein D, Burns T, Steimle JD, Yang XH, Moskowitz IP, Hurles M, Lifton RP, Nickerson D, Bamshad M, Eichler EE, Mital S, Sheffield V, Quertermous T, Gelb BD, Portman M, Ashley EA** 2016 De Novo and Rare Variants at Multiple Loci Support the Oligogenic Origins of Atrioventricular Septal Heart Defects. PLoS genetics 12:e1005963
47. **Glessner JT, Bick AG, Ito K, Homsy J, Rodriguez-Murillo L, Fromer M, Mazaika E, Vardarajan B, Italia M, Leipzig J, DePalma SR, Golhar R, Sanders SJ, Yamrom B, Ronemus M, Iossifov I, Willsey AJ, State MW, Kaltman JR, White PS, Shen Y, Warburton D, Brueckner M, Seidman C, Goldmuntz E, Gelb BD, Lifton R, Seidman J, Hakonarson H, Chung WK** 2014 Increased frequency of de novo copy number variants in congenital heart disease by integrative analysis of single nucleotide polymorphism array and exome sequence data. Circulation research 115:884-896
48. **Warburton D, Ronemus M, Kline J, Jobanputra V, Williams I, Anyane-Yeboa K, Chung W, Yu L, Wong N, Awad D, Yu CY, Leotta A, Kendall J, Yamrom B, Lee YH, Wigler M, Levy D** 2014 The contribution of de novo and rare inherited copy number changes to congenital heart disease in an unselected sample of children with conotruncal defects or hypoplastic left heart disease. Human genetics 133:11-27
49. **Keitges EA, Pasion R, Burnside RD, Mason C, Gonzalez-Ruiz A, Dunn T, Masiello M, Gebbia JA, Fernandez CO, Risheg H** 2013 Prenatal diagnosis of two fetuses with deletions of 8p23.1, critical region for congenital diaphragmatic hernia and heart defects. American journal of medical genetics. Part A 161A:1755-1758
50. **Geng J, Picker J, Zheng Z, Zhang X, Wang J, Hisama F, Brown DW, Mullen MP, Harris D, Stoler J, Seman A, Miller DT, Fu Q, Roberts AE, Shen Y** 2014 Chromosome microarray testing for patients with congenital heart defects reveals novel disease causing loci and high diagnostic yield. BMC genomics 15:1127
51. **Blinder JJ, Martinez HR, Craigen WJ, Belmont J, Pignatelli RH, Jefferies JL** 2011 Noncompaction of the left ventricular myocardium in a boy with a novel chromosome 8p23.1 deletion. American journal of medical genetics. Part A 155A:2215-2220
52. **Tomita-Mitchell A, Mahnke DK, Struble CA, Tuffnell ME, Stamm KD, Hidestrand M, Harris SE, Goetsch MA, Simpson PM, Bick DP, Broeckel U, Pelech AN, Tweddell JS, Mitchell ME** 2012 Human gene copy number spectra analysis in congenital heart malformations. Physiological genomics 44:518-541
53. **Guimiot F, Dupont C, Fuentes-Duarte A, Aboura A, Bazin A, Khung-Savatovsky S, Tillous-Borde I, Delezoide AL, Azancot A** 2013 Maternal transmission of interstitial 8p23.1 deletion detected during prenatal diagnosis. American journal of medical genetics. Part A 161A:208-213
54. **Osoegawa K, Iovannisci DM, Lin B, Parodi C, Schultz K, Shaw GM, Lammer EJ** 2014 Identification of novel candidate gene loci and increased sex chromosome aneuploidy among infants with conotruncal heart defects. American journal of medical genetics. Part A 164A:397-406
